# Aging Induces Hepatic Oxidative Stress and Nuclear Proteomic Remodeling in Liver from Wistar Rats

**DOI:** 10.3390/antiox10101535

**Published:** 2021-09-27

**Authors:** Brenda Bárcena, Aurora Salamanca, Cristina Pintado, Lorena Mazuecos, Margarita Villar, Eduardo Moltó, Elena Bonzón-Kulichenko, Jesús Vázquez, Antonio Andrés, Nilda Gallardo

**Affiliations:** 1 Biochemistry Section, Regional Center for Biomedical Research (CRIB), Faculty of Sciences and Chemical Technologies, University of Castilla-La Mancha, Avda. Camilo Jose Cela 10, 13071 Ciudad Real, Spain; brenda.barcena@alu.uclm.es (B.B.); aurora.Salamanca@alu.uclm.es (A.S.); lorena.mazuecos@uclm.es (L.M.); antonio.andres@uclm.es (A.A.); 2 Biochemistry Section, Regional Center for Biomedical Research (CRIB), Faculty of Environmental Sciences and Biochemistry, University of Castilla-La Mancha, Avda. Carlos III s/n, 45071 Toledo, Spain; cristina.pintado@uclm.es (C.P.); eduardo.molto@uclm.es (E.M.); 3 SaBio, Instituto de Investigación en Recursos Cinegéticos IREC-CSIC-UCLM-JCCM, Ronda de Toledo s/n, 13005 Ciudad Real, Spain; 4 Cardiovascular Proteomics Laboratory, Centro Nacional de Investigaciones Cardiovasculares Carlos III and CIBER de Enfermedades Cardiovasculares (CIBERCV), 28029 Madrid, Spain; ebonzon@cnic.es (E.B.-K.); jvazquez@cnic.es (J.V.)

**Keywords:** oxidative stress, aging, aging-associated chronic diseases, liver proteome, RNA splicing

## Abstract

Aging is a continuous, universal, and irreversible process that determines progressive loss of adaptability. The liver is a critical organ that supports digestion, metabolism, immunity, detoxification, vitamin storage, and hormone signaling. Nevertheless, the relationship between aging and the development of liver diseases remains elusive. In fact, although prolonged fasting in adult rodents and humans delays the onset of the disease and increases longevity, whether prolonged fasting could exert adverse effects in old organisms remains incompletely understood. In this work, we aimed to characterize the oxidative stress and nuclear proteome in the liver of 3-month- and 24-month-old male Wistar rats upon 36 h of fasting and its adaptation in response to 30 min of refeeding. To this end, we analyzed the hepatic lipid peroxidation levels (TBARS) and the expression levels of genes associated with fat metabolism and oxidative stress during aging. In addition, to gain a better insight into the molecular and cellular processes that characterize the liver of old rats, the hepatic nuclear proteome was also evaluated by isobaric tag quantitation (iTRAQ) mass spectrometry-based proteomics. In old rats, aging combined with prolonged fasting had great impact on lipid peroxidation in the liver that was associated with a marked downregulation of antioxidant genes (*Sod2*, *Fmo3*, and *Cyp2C11*) compared to young rats. Besides, our proteomic study revealed that RNA splicing is the hepatic nuclear biological process markedly affected by aging and this modification persists upon refeeding. Our results suggest that aged-induced changes in the nuclear proteome could affect processes associated with the adaptative response to refeeding after prolonged fasting, such as those involved in the defense against oxidative stress.

## 1. Introduction

Aging is a natural process involving the whole body. As a natural process, Gems [1] has suggested that aging is not a disease, even though older people do tend to get ill more often and to develop serious and chronic diseases. Genomic instability, telomere wasting, epigenetic alterations, loss of proteostasis, dysregulated nutrient sensing pathways, mitochondrial dysfunction, cellular senescence, stem cell depletion, and altered intercellular communication have emerged as the nine hallmarks of aging [2]. All of them are triggered by a myriad of stress conditions and involve important risk factors for metabolic and physiological disabilities. Many studies in experimental models and humans have been conducted to find the link between oxidative stress and aging at the molecular and cellular levels and revealed that in conditions of metabolic syndrome (MS), oxidative stress could accelerate aging [3]. Moreover, a considerable amount of evidence points to the process of immunosenescence as the major contributor to the chronic basal inflammation associated with aging (inflammaging) and thereby to increased oxidative stress [4,5]. Nevertheless, the biology of aging continues to be poorly understood and whether oxidative stress is a pivotal regulator of aging and age-associated diseases remains conflicting and needs to be resolved.

Metabolic syndrome (MS) is an insulin-resistant state associated with obesity and common in aging. In this condition, fat is redistributed and deposited in non-adipose tissues, including the liver. In addition, oxidative stress, assessed by lipid oxidation, is increased, whereas systemic antioxidant defense capacity is reduced [6].

Non-alcoholic fatty liver disease (NAFLD) encompasses the entire spectrum of fatty liver diseases occurring in the absence of secondary causes and ranging from non-alcoholic fatty liver (NAFL) to non-alcoholic steatohepatitis (NASH). The prevalence and severity of NAFLD in the general population increases with age and enhances the risk of developing type 2 diabetes mellitus (T2D) and cardiovascular diseases. Even though the mechanisms of progression of NAFLD from simple steatosis to steatohepatitis (NASH), fibrosis, cirrhosis, and hepatocellular carcinoma have been extensively documented [7], it needs to be completely elucidated.

In mammals, the liver plays an important role in lipid metabolism. Lipid deposition activates several cellular stress pathways, including oxidative stress and endoplasmic reticulum (ER) stress, producing insulin resistance and inflammation. Increased production of free radicals that is not counterbalanced by adequate antioxidant defenses induces lipid peroxidation that further proceeds with radical chain reaction and advanced glycation end-products (AGEs). Moreover, peroxidized lipids and AGEs induce immune responses in steatotic livers and accelerate the progression to steatohepatitis and cirrhosis and ultimately to hepatocellular carcinoma [8,9,10].

The aged liver also manifests structural and functional changes in the cellular nucleus. Age-dependent changes in nucleosome occupancy have been linked to the development of steatosis in aged liver [11]. Oxidative stress can accelerate telomere shortening and senescence in fibrotic livers [12] and chromatin disorganization at the nuclear lamina have been associated with altered Foxa2 binding, de-repression of lipogenic genes, and hepatic steatosis [13]. Moreover, impaired nucleo-cytoplasmic transport is considered as a fundamental pathological factor in aging diseases [14]. Despite this knowledge, the current understanding of the effects of aging on the hepatic nuclear biological processes is scarce. The old Wistar rat is a physiological model of aging with metabolic disorders like those observed in the human MS, including increases in visceral fat, dyslipidemia, and insulin resistance. In addition, and closely related to dyslipidemia and insulin resistance, the aged Wistar rat manifests adipose tissue inflammation and liver steatosis and fibrosis [15,16,17,18]. Most of our knowledge about the molecular changes that occur in the liver of Wistar rats with aging comes from studies of gene expression and protein distribution patterns [16]. In this regard, we published that aging causes a significant increase in the mRNA abundance of lipogenic transcription factors and enzymes, such as carbohydrate-responsive element-binding protein (ChREBP), diacylglycerol acyltransferases 1 and 2 (DGAT1/2), and microsomal triglyceride transfer protein (MTTP), whereas the mRNA levels of the forkhead transcription factor Foxa2 and the most important enzyme associated with mitochondrial fatty acid oxidation carnitine-palmitoyl transferase-1 (CPT-1a) were markedly decreased in the liver of old Wistar rats [16,17]. Contrary to what was observed in young rats, lipogenic ChREBP was enriched in the nuclear fraction of liver homogenate from old rats under 36 h fasting, whereas oxidative Foxo1 and Foxa2 were enriched in the cytoplasmic fraction [16]. These results indicate that nucleocytoplasmic shuttling in response to the fasting-refeeding cycle is impaired in the liver of old rats, causing inefficient nucleocytoplasmic communication that might affect transcription, and the management of lipid metabolism and oxidative stress [19,20]. Nevertheless, the mechanisms that could deregulate hepatic nucleocytoplasmic distribution during aging are currently unknown. Notably, high-fat diet (HFD) also impaired the nucleo-cytoplasmic distribution of the nuclear receptor HNF4α in steatotic livers from mice, which was associated with increased hepatic oxidative stress [21].

These observations are consistent with the finding that certain splicing machinery components are severely dysregulated in the liver of patients with obesity and liver steatosis and in animal models of NAFLD and NASH [22,23,24,25]. In this regard, other findings have demonstrated the contribution of alternative splicing of pre-mRNAs to transcriptome diversity in conditions of oxidative stress [26,27,28].

However, the effects of aging on the mRNA alternative splicing machinery are poorly understood. Therefore, we hypothesized that a significant part of aging-mediated liver damage in Wistar rats can be attributed to alterations in gene expression derived from disturbed alternative mRNA splicing that could modify hepatic cellular function and predispose to liver damage and disease. In addition, we tested the hypothesis that the hepatic nuclear processes affected more by aging are present in both the fasted and the refed state.

Hence, we measured in young and old rats the liver levels of lipid peroxidation (TBARS) for estimation of the oxidative status, and the mRNA levels of antioxidant and proinflammatory enzymes and cytokines. To better understand how the liver of old rats responds to oxidative stress, the rats were challenged with a prolonged fast-refeeding cycle. Contrary to what happens with caloric restriction, prolonged fasting decreases the antioxidant capacity of liver cells and increases the sensitivity of fat to oxidative damage because it causes a rearrangement of lipid double bonds [29,30]. Although data from experimental and observational studies in rodents and humans, respectively, suggested that both prolonged fasting and periodic fasting cycles have the ability to delay the onset of disease and increase longevity [31], prolonged fasting could exert adverse effects in aged organisms with multiple age-related diseases and this needs to be investigated.

We further applied a proteomic analysis by isobaric tag quantitation (iTRAQ) to elucidate how aging affects the hepatic nuclear proteome. This sub-cellular fractionation allowed more in-depth analysis of the proteome and the identification of some nuclear and perinuclear proteins that are not easily detected in total extracts due to the complexity of the sample [32]. We applied a prolonged fasting-refeeding paradigm to assess the extent to which the nuclear proteome is modified under these conditions in old compared with young rats.

In this study, we show that the liver from old rats under prolonged fasting has significantly higher levels of TBARS, reduced expression of antioxidant genes, and enhanced expression of markers of ER stress and inflammation, in agreement with previous results [33,34]. Consistent with this, we show a profound remodeling of the hepatic nuclear proteome in aged Wistar rats compared with young animals. The changing proteins are mainly involved in nucleosome assembly, chromatin remodeling, RNA processing and splicing, spliceosomal complex structure, ribonucleoprotein complex, DNA synthesis, DNA damage and repair, nuclear export/import, cell cycle, nuclear envelope organization, and nucleoplasm organization. Of note, the most affected nuclear process in aged rats is the alternative RNA splicing, being affected by several components of the splicing process. Our results also show alterations of many of the proteins involved in the mitochondrial metabolic process, endoplasmic reticulum process, and the defense against oxidative stress damage. Taken together, these findings provide novel insights into the molecular changes induced by aging in the liver of Wistar rats that could aid in understanding the pathogenesis of NAFLD. Finally, quantitative proteomics analysis revealed a different adaptive response to the fasting/refeeding approach in aged rats compared to the young animals.

## 2. Materials and Methods

### 2.1. Animals and Ethic Statements

The experiments were performed in male 3- and 24-month-old Wistar rats from our in-house colony (Centre of Molecular Biology, Madrid, Spain). The maximal life span of male Wistar rat is about 32–34 months, while the mean life span is about 24 months [35]. Thus, the 24-month-old rats used in the present study were middle-old age animals. These old rats were not at high risk of mortality and did not present apparent signs of frailty [15,16,17,36], although they showed higher intracellular accumulation of lipofuscin, compared to 3-month-old Wistar rats [17], a marker of cellular senescence.

Animals were housed in climate-controlled quarters with a 12-h light cycle. All rats in this study were fed a standard chow diet (2014 Teklad Global 14% Protein Rodent Maintenance Diet) from Harlan Laboratories and water. Animals were handled according to the European Union laws (2010/63/EU) and following the Spanish regulations (RD 53/2013) for the use of laboratory animals. The experimental protocols were approved by the Institutional Scientific Committee of Bioethics under project license CE/99-1835-A308. All efforts were made to minimize animal suffering and to reduce the number of animals used.

Animals were randomly divided into three groups, each constituted by four 3-month- and four 24-month-old rats. Animals of the first group were fasted (nutrient withdrawal) 16 h before euthanizing, those of the second group were fasted (nutrient withdrawal) 36 h before euthanizing, and those of the third group were fasted for 36 h and then refed for 30 min before euthanizing. The third group was introduced for the purpose of evaluating the adaptation for the fed state following prolonged fasting. Rats were anesthetized by CO_2_ inhalation and sacrificed by decapitation at 09:30 AM.

### 2.2. Analytical Procedures

Blood was obtained immediately after fasting (16 or 36 h) in the first and second group and after 30 min of refeeding in the third group. Serum glucose was measured immediately using an Accutrend Glucose Analyzer (Roche Diagnostics Corp., Indianapolis, IN, USA). Serum triacylglycerides (TAG) and nonesterified fatty acid (NEFA) contents were quantified by specific enzymatic kits from Wako Chemicals (Neuss, Germany). Total-cholesterol and cholesterol-HDL (high-density lipoprotein) levels were measured, respectively, using an enzymatic kit from Stanbio Laboratory (Boerne, TX, USA). Insulin and leptin levels were assayed using specific rat ELISA kits from Spi-Bio (Montigny le Bretonneaux, France) and the levels of total ketone bodies and glucagon were determined using an Autokit Total Ketone Bodies and an ELISA glucagon kit, respectively, both from WAKO, Chemical Neus. Ghrelin (acetylated and unacetylated) levels were assayed in plasma using specific rat ELISA kits from Spi-Bio (Montigny le Bretonneaux, France) according to the manufacturer’s instructions.

Liver and visceral fat depots were carefully dissected and weighed. Then, tissues were flash frozen in liquid nitrogen and stored at −70 °C until used. Frozen liver samples were used for glycogen and TAG measurement. Neutral lipids were extracted from the liver as previously described [37] and the hepatic TAG content was analyzed by the enzymatic kits from Stanbio Laboratory (Boerne, TX, USA). Glycogen levels were assessed in the liver using a glycogen assay kit II (ab 169558, Abcam, Boerne, TX, USA) following the manufacturer’s instruction. Both TAG and glycogen were measured in triplicate and both contents were expressed as mg/g wet tissue.

### 2.3. Total Extract from Liver and Immunoblot Analysis

A piece of fresh liver was thawed, cut into small pieces on ice, and suspended (4 mL buffer/g tissue) in cold Krebs-Henseleit buffer pH 7.4 (116 mM NaCl, 4.7 mM KCl, 1.2 mM CaCl_2_, 1.2 mM KH_2_PO_4_, 1.2 mM MgSO_4_.7H_2_O, 5.5 mM glucose, 25 mM NaHCO_3_, 1 mM PMSF, 10 µg/mL leupeptin, 1 µg/mL pestatin, 2 mM NaF, 1 mM Na_3_VO_4_) before homogeneization with 10 passes of a loose-fitting B pestle in a Dounce homogenizer. Then, the homogenates were incubated for 1 h at 4 °C and centrifuged at 800× *g* for 15 min at 4 °C. The supernatant (total extract) was collected and frozen at −70 °C until use.

Protein content of the mitochondrial oxidative phosphorylation OXPHOS complex was determined with Total OXPHOS rodent WB antibody cocktail (6 µg/mL, ab110413, Abcam, Cambridge, UK), which contain 5 mouse monoclonal antibodies, one each against CI subunit NDUFB8, CII-30kDa, CIII-Core protein, CIV subunit I, and CV alpha subunit of OXPHOS. The antibody cocktail was used according to the manufacturer’s instructions. In total, 20 μg of protein were separated under reducing conditions on 12.5% SDS-PAGE, transferred to nitrocellulose sheets (0.2 μm) (Bio-Rad, Madrid, Spain), and incubated overnight at 4 °C with the antibody cocktail followed by incubation at room temperature for 30 min with secondary antibody conjugated with horseradish peroxidase. To ensure the specificity of OXPHOS complexes-immunoreactive protein, rat liver mitochondria Western Blot control was used as a positive control (data not shown). Primary antibody anti-β-actin (1:1000, ab8226) from Abcam, Cambridge, UK, was used as the control for protein loading. The secondary antibody used was goat anti-mouse conjugated with horseradish peroxidase (1:5000, 170-6516) from Bio-Rad, Spain. Blots were repeated 3 times to assure the reproducibility of the results. The immunocomplexes formed were visualized using the ECL Western-blotting detection kit (Amersham Biosciences, Inc., Piscataway, NJ, USA) and the images were subjected to a densitometric analysis with a G-Box Densitometer, and bands were quantified by scanning densitometry with the exposure in the linear range using Gene Tools software (Syngene, Cambridge, UK).

### 2.4. Separation of Rat Liver Nuclear Enriched Fraction

To obtain the hepatic nuclear-enriched fraction (NEF), we followed the protocol described by [38]. Two different buffers were used: HLB buffer (10 mM HEPES pH 7.4, 1.5 mM MgCl_2_, 10 mM KCl, 1 mM DTT, 1 mM PMSF, 10 µg/mL leupeptin, 1 µg/mL pepstatin, 2 mM NaF, 1 mM Na_3_VO_4_) and NLB buffer (10 mM HEPES pH 7.9, 100 mM KCl, 3 mM MgCl_2_, 0.1 mM EDTA, 1 mM DTT, 1 mM PMSF, 10 µg/mL leupeptin, 1 µg/mL pepstatin, 2 mM NaF, 1 mM Na_3_VO_4_). Liver samples were homogenized using a manual Dounce homogenizer in buffer HLB (500 µL /100 mg tissue), then they were incubated for 5 min on ice with 10% Igepal, to prevent the break of the nucleus. The homogenate was vigorously stirred for 30 s and centrifuged at 10,500× *g* for 30 min at 4 °C. The supernatant (cytosolic fraction) was removed. The pellet was resuspended in 500 µL of NLB buffer and incubated for 30 min at 4 °C. Then, 1/10 volume of 4M (NH_4_)_2_SO_4_ was added and the mixture was incubated for 30 min at 4 °C. Finally, the homogenate was centrifuged for 10 min at 16,000× *g* at 4 °C, the pellet was discarded, and the supernatant (NEF) stored at −70 °C until used.

### 2.5. RNAE and Real-Time RT-PCR

Total RNA was isolated from liver using the Trizol reagent (Invitrogen) following the manufacturer’s instructions. The cDNA was synthesized from 1.5 μg of DNase-treated RNA by using the reverse-transcriptase activity from Moloney murine leukaemia virus (Gibco-BRL), and p[dN]6 (Boehringer Mannaheim, Germany) as a random primer. Relative quantitation of superoxide dismutase 2 (*Sod2*), stearoyl-CoA desaturase 1 (*Scd-1*), flavin-containing dimethylaniline monoxygenase 3 (*Fmo3*), cytochrome P450 monooxygenase isoforms 2c11 (*cyp2c11*), 78-kDa glucose-regulated protein (*Grp78*), protein disulfide isomerase (*Pdi*), interleukin 6 (*Il-6*), Interleukin 10 (*Il-10*), and tumor necrosis factor alpha (*Tnfα*) expression were measured using Pre-Developed TaqMan Assay Reagents (PE Applied Biosystem). Quantitative PCR was performed on an ABI PRISM 7500 Fast Sequence Detection System instrument and software (PE Applied Biosystem, Foster City, CA, USA). To standardize the amount of sample cDNA added to the reaction, amplification of endogenous control 18SrRNA was included in a separate well using VIC (TaqMan Assay) as the real-time reporter. The ΔΔCT method was used to calculate the relative differences between experimental conditions and control groups as fold change in gene expression. Details about the genes used in this study are provided in the Supplementary Table S1.

### 2.6. TBARS Determination

Lipid peroxidation was analyzed by the measurement of thiobarbituric acid reactive substances (TBARSs) in LTE. For this purpose, we added 600 µL of 0.5% TBA (thiobarbituric acid, Merck, Darmstadt, Germany), prepared in 20% acetic acid (pH 3.5), to 50 µL of the liver homogenate. The mix was incubated 1 h at 90 °C for adduct formation between the TBA and lipid peroxides. Then, it was incubated for 5 min in ice prior to the addition of 50 μL of 10% SDS. After, the mixture was centrifuged at 500× *g*, at room temperature (RT) for 15 min. Optical density (OD) was read at 532 nm and TBARS levels were calculated using a standard curve of malondialdehyde bis-dimethyl acetal. TBARS concentration was expressed as nmol/mL per mg of tissue.

### 2.7. Calculations and Statistical Analysis

Visceral adiposity was the sum of the weight in grams of epidydimal and retroperitoneal fat pads. Statistical analysis was performed using the GraphPad Prism version 8.2 for Windows (GraphPad Software). Data are presented as the mean ± SEM of 4 rats per group and age. Significant differences among groups, both when comparing the effects of fasting duration (16 and 36 h) in 3- and 24-month-old Wistar rats and when comparing the effects of refeeding after a 36-h fasting in 3- and 24-month-old Wistar rats, were determined by two-way ANOVA, followed by Tukey’s post hoc test, to detect the main effects of age, fasting-refeeding, and their interaction. Statistical significance was set at *p* ≤ 0.05. Correlation analysis was determined by Pearson’s correlation coefficient test (r).

### 2.8. Protein Extraction, iTRAQ Labelling, Proteomics Data Acquisition, and Analysis

Proteins from the liver NEF of young-mature (*n* = 4) and old Wistar rat (*n* = 4), either fasted for 36 h or fasted 36 h and then refed for 30 min before euthanizing, were extracted by tissue homogenization with ceramic beads (MagNa Lyser Green Beads apparatus, Roche, Germany) in extraction buffer (50 mmol/L Tris-HCl, 1 mmol/L EDTA, 4% SDS, pH 8.5). The protein extracts (100 µg from each sample) were in-gel digested [39], and the resulting peptides were labelled with iTRAQ4-plex following the manufacturer´s instructions. The labelled peptides were analyzed by nano-liquid chromatography-tandem mass spectrometry (nanoLC-MS/MS) using a quadrupole ion trap-orbitrap ELITE mass spectrometer (Thermo Scientific).

Peptide and protein identification were performed using the SEQUEST algorithm integrated in Proteome Discoverer 1.3 (Thermo Scientific). MS/MS scans were searched against a joined rat and mouse target database (UniProtKB/Swiss-Prot, November 2011, 125669 protein sequences). Met oxidation (15.994915 Da) was set as variable modification, while Cys carbamidomethylation (57.021464 Da) and iTRAQ4-plex (144.102063 Da) on Lys and peptide N-terminus were used as fixed modifications.

Precursor mass tolerance was set to 10 ppm and fragment mass tolerance at 0.03 Da; precursor charge range was set to 2–4; and 3 was the maximum fragment charge. Two miss-cleavages were allowed and only y- and b-ions were used for scoring. SEQUEST results were analyzed using the probability ratio method [40] and false discovery rates (FDR) of peptide identifications were calculated from the search results against the inverted databases using the refined method [41,42]. Protein abundance changes from MS/MS spectra of all the identified peptides with an FDR lower than 5% were analyzed using the Generic Integration Algorithm [43] on the basis of the WSPP model [44]. The biological interpretation of the results was made using the Systems Biology Triangle (SBT) as described [43]. The Gene Ontology, KEGG, and REACTOME databases were used. To analyze the effect of aging and/or the nutritional condition on the hepatic NEF proteome, we performed the following comparisons: (a) effects of 36 h fasting in young and old rats, (b) effects of 30 min refeeding after 36 h fasting in young and old rats, (c) effect of fasting/refeeding in young rats, and (d) effect of fasting/refeeding in old rats. Functional protein analysis is presented as the protein log_2_-ratios between the four comparisons mentioned above standardized according to their estimated variances (z_q_ values, see the Supplementary Table S4) classified in terms of the Gene Ontology Biological Process. The mass spectrometry raw proteomics data have been deposited to the Proteome X Change consortium data set identifier PXD027773.

An overview of the methods and procedures employed in this work is shown in the Supplementary Figure S1.

## 3. Results 

### 3.1. Effect of Fasting or Fasting/Refeeding on Metabolic Characteristics of Young and Old Wistar Rats

The main goal of this work was to gain insight into the process of aging in Wistar rats. We focused on the liver because the prevalence of chronic liver diseases, such as NAFLD and NASH, is increased in the elderly population. First, we wanted to analyze the effect of fasting on different metabolic parameters in young and old Wistar rats sacrificed after 16 h and/or 36 h fasting (Table S2). As expected, body weight, liver weight, liver TAG, and visceral adiposity were higher in 24-month- compared with 3-month-old Wistar rats. BW was not modified after 16 h or after 36 h of fasting in both groups of rats. Food deprivation for 36 h decreased insulinemia in young rats. On the contrary, insulinemia was increased in old rats following 36 h of fasting, according to their insulin-resistant state [15,16,17,18]. As previously reported [15,16,17,18], no differences were observed between 3- and 24-month-old Wistar rats with respect to serum glucose and NEFA concentration after 16 or 36 h of fasting. NEFA concentrations decreased to a similar extent in both groups of rats after 36 h of fasting. Nevertheless, the increase in ketone bodies in response to prolonged fasting was diminished in 24-month-old rats as reported [16]. Liver weight and liver TAG were higher at 16 and 36 h of fasting in 24-month- compared with 3-month-old rats. On the other hand, prolonged fasting decreased the liver weight but significantly increased the hepatic TAG content in old rats. In contrast, the hepatic weight and TAG content tended to decrease in young rats in response to prolonged fasting. In addition, prolonged fasting markedly increased the already high hepatic TBARS levels found in 16-h-fasted old rats, while the content of hepatic TBARS upon prolonged fasting in young rats reached a similar level to that found in 16 h fasted old rats (Supplementary Table S2). In summary, results from the Supplementary Table S2 confirm our previous studies [16] and data from humans showing elevated circulating ketone bodies after prolonged fasting periods (36 h) [45], suggesting that following 36 h of fasting, there was a perceptible metabolic transition from utilizing carbohydrates and glucose to fatty acids and ketone bodies as the major cellular fuel sources in both old and young animals.

Having established that prolonged fasting (36 h) exacerbated steatosis and liver oxidative stress in 24-month-old rats, we decided to assess whether 36 h of fasting followed by a short period of refeeding might accelerate oxidative damage in the aged liver and evaluate their ability to respond quickly to nutrient availability. To this end, we first analyzed the responses of hormones and metabolites to this fasting-refeeding cycle. Furthermore, we also assessed the relationships between the expression of genes encoding for metabolic enzymes involved in the regulation of redox homeostasis with the levels of lipid peroxidation in liver. Finally, we studied the effects of the combination of aging and prolonged fasting on the hepatic nuclear proteome by iTRAQ quantitative proteomics in young and old Wistar rats under two physiological conditions: following 36 h of fasting or after 36 h of fasting and then refeeding for 30 min.

The responses to prolonged fasting-refeeding in 3- and 24-month-old Wistar rats are illustrated in Table 1. Our results indicate that both groups of rats were able to maintain normoglycemia after prolonged fasting (36 h). Aged rats showed higher levels of insulinemia, glucagonemia, and leptinemia compared with the young ones, even after a prolonged fasting state. After refeeding, a condition that changes the levels of glucose, insulin and glucagon, glucose, and liver glycogen contents increased significantly only in 3-month-old rats (Table 1). Interestingly, in these rats, we observed a strong insulin response to nutrient availability while in old rats, the insulin response was replaced by the glucagon response (Table 1).

We further measured serum lipid profiles and hepatic fat deposition. Under both conditions (fasting and fasting/refeeding) and consistent with previous reports [16,17,46], serum and hepatic TAG levels were markedly higher in old compared with young rats (Table 1).

Furthermore, serum levels of the liver enzyme alanine aminotransferase (ALT) and the marker of systemic inflammation C-reactive protein (CRP) were also significantly elevated in old rats (Table 1). Thus, our results confirm that aging induces hepatic TAG accumulation in the Wistar rat. Moreover, and like previous findings obtained in 16-h-fasted rats [16], we noticed that levels of total ketone bodies (TKBs) were lower in older than in younger rats after 36 h of fasting (Table 1), suggesting reduced synthesis of ketone bodies in the liver from old rats, a result that was further confirmed by proteomics. As shown in Table 1, refeeding immediately inhibits hepatic ketogenesis in both groups of rats as deduced by the decline in serum total ketone bodies levels (TKB) (Table 1). Interestingly, refeeding increased serum NEFA levels in old rats, consistently with a state of insulin resistance that persists even after refeeding for 3 h as we have previously published [16].

In addition, we showed significant interactions of the fasting-refeeding cycle with age for serum insulin, glucagon, NEFA, TKB, and liver glycogen (Table 1). We further measured serum acetylated and unacetylated ghrelin, due to its role in the regulation of systemic energy metabolism and redox homeostasis in the liver. There was a decrease, albeit not statistically significant at *p* ≤ 0.05, in the levels of unacetylated ghrelin (total ghrelin) in old rats compared with those of young and lean rats after 36 h of fasting (Table 1). Decreased levels of unacetylated ghrelin have been observed in obese rats with hepatic steatosis [47]. Acetylated ghrelin and the acetylated/unacetylated ghrelin ratio were augmented by aging in Wistar rats under prolonged fasting (Table 1). Taken together, our results indicate prolonged fasting induces different metabolic reprograming in aged rats compared with their young counterparts.

### 3.2. Changes in Hepatic Lipid Peroxidation Levels and in the Expression Levels of Genes Involved in Lipid Metabolism and Oxidative Stress during Aging

**Figure 1:**
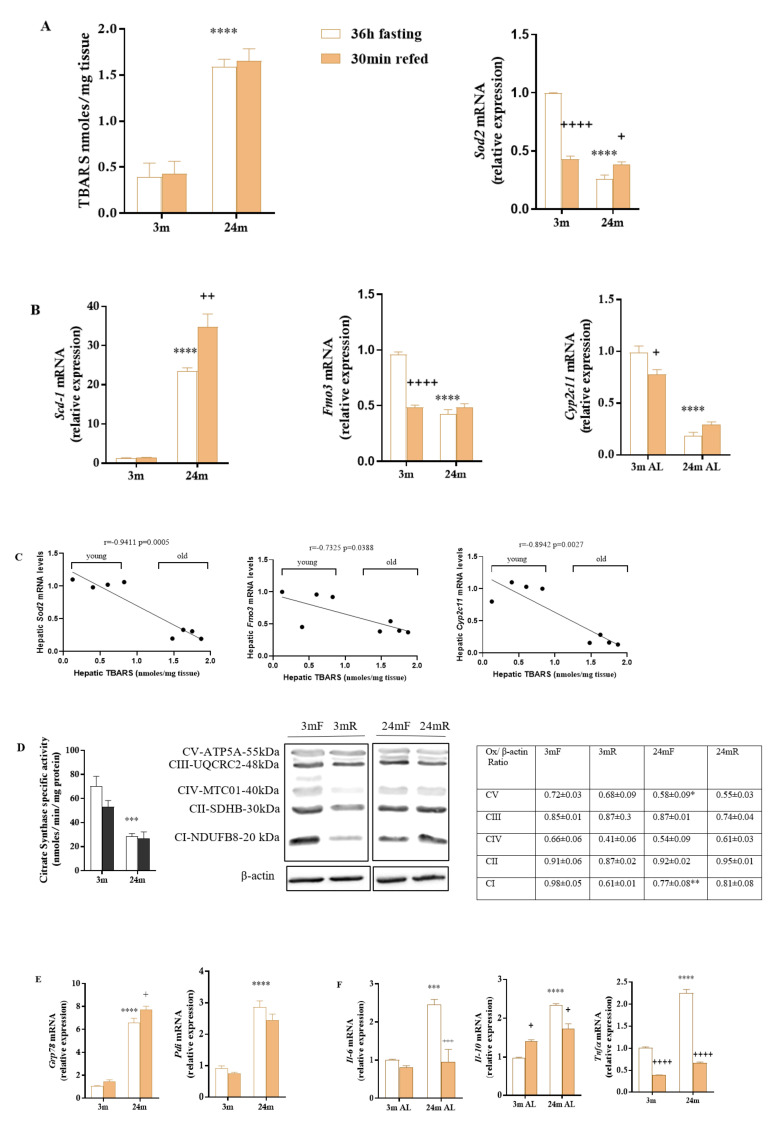
Thiobarbituric acid reactive substance (TBARS) levels and mRNA levels of the antioxidant gene *Sod2* (**A**), mRNA levels of the oxidoreductase genes *Scd1*, *Fmo3*, and *Cyp2c11*c (**B**), correlation analysis between TBARS levels and *Sod2*, *Fmo3* and *Cyp2c11* mRNA levels in Wistar rat after prolonged fasting (**C**), hepatic citrate synthase activity and OXPHOS protein complex levels (**D**), mRNA levels of genes implicated in ER stress (*Grp78* and *Pdi*) (**E**), and the mRNA levels of the proinflammatory (*Il-6* and *Tnfα*) and anti-inflammatory (*Il-10*) cytokines (**F**), in the liver of Wistar rats during a fasting-refeeding cycle. Values are expressed as means ± SEM of 4 animals. Data were analyzed by two-way ANOVA followed by Tukey’s correction. Correlation analysis was determined by Pearson’s correlation coefficient test (r). Two-way ANOVA was performed to detect main effects of age, fasting-refeeding, and age fasting-refeeding interaction. *** *p* < 0.001, **** *p* < 0.0001 vs. the young rats. ^+^ *p* < 0.05, ^++^ *p* < 0.01, ^+++^ *p* < 0.001, ^++++^ *p* < 0.0001 vs. the age-matched fasted rats. Two-way ANOVA indicate a significant effect of age on *Grp78* (*p* < 0.0001; F = 305.4; Df = 1) and *Pdi* (*p* < 0.0001; F = 13.26; Df = 1). Two-way ANOVA indicated a significant interaction between fasting-refeeding and age for *Sod2* (*p* < 0.0001; F = 185.8; Df =1); *Scd-1* (*p* < 0.0078; F = 10.15; Df = 1); *Fmo3* (*p* < 0.0001; F = 71.68; Df = 1); *Cyp2c11* (*p* = 0.0041; F = 12.53; Df = 1); *Il-6* (*p* < 0.0035; F = 13.11; Df = 1); *Il-10* (*p* < 0.0001; F = 83.02; Df = 1) and *Tnfα* (*p* < 0.0001; F = 136.6; Df = 1).

We have previously reported that ROS accumulate in the liver of aged Wistar rats [15]. In this regard, lipofuscin, a marker of aging that reveals oxidative stress, is also accumulated [15,17,48]. To examine the effects of ROS on lipid peroxidation damage, ER stress, and inflammation, we first measured the levels of TBARS and the mRNA levels of *Sod2*, a gene involved in the management of oxidative stress. TBARS were consistently higher in the liver of old Wistar rats (Figure 1A), suggesting an increment in lipid peroxidation damage that correlates with reduced expression of the antioxidant *Sod2* (Figure 1A) consistent with previous studies [49]. To evaluate the contribution of oxidative metabolism to fat accumulation and increased levels of peroxidated lipids in old rats, we measured the mRNA levels of three oxidoreductases: *Scd1*, a key regulatory enzyme in the biosynthesis of monounsaturated fatty acids (MUFAs) that promotes hepatic fat accumulation; *Fmo3*, involved in microsomal fatty acid ω-oxidation, xenobiotic metabolism, and protection against oxidative and ER stress; and *Cyp2c11*, involved in hormone, xenobiotic oxidation, and arachidonic/linoleic acid metabolism. The mRNA levels of *Scd-1* increased in the liver from old rats compared to the control group, indicating a high capacity for TAG synthesis and accumulation (Figure 1B). As expected, hepatic Fmo3 and *Cyp2c11* are downregulated in older rats (Figure 1B), proving that in aged liver, peroxisome and microsome fatty acid oxidation and the defense capacity against oxidative stress is impaired. Those results were also confirmed by quantitative proteomics (Supplementary Table S3).

Figure 1C shows that hepatic TBARS levels correlate negatively with the hepatic expression of *Sod2*, *Fmo3*, and *Cyp2c11*, indicating that peroxisome and microsome fatty acid oxidation has the capacity to impact on the levels of peroxidated lipids in the liver of Wistar rats (Figure 1C). Analysis of the effects of the fasting-feeding cycle showed that *Scd-1* increased after refeeding in old rats (Figure 1B), supporting fat deposition in the liver. On the contrary, *Fmo3* and *Cyp2c11*, the mRNA levels of which decreased after refeeding in young rats, remained unchanged in the liver of old rats (Figure 1B). Collectively, these results imply that the fasting-feeding cycle could be involved in increased oxidative stress in aged liver as has been previously suggested [50,51,52,53].

Aging and oxidative stress alters the mitochondrial process. Figure 1D shows that hepatic citrate synthase activity and the levels of subunits of the mitochondrial OXPHOS complex I and V decreased with aging (Figure 1D). Proteomic analysis also corroborated these results (Supplementary Table S3).

Aging, starvation, and increased ROS can also cause unfolded or misfolded proteins to accumulate in the endoplasmic reticulum (ER), initiating an unfolded protein response (UPR) that reduces protein translation, increases inflammation, and impairs proteostasis. The final consequence is the accumulation of damaged proteins and undegradable aggregates, such as lipofuscin [54,55]. Figure 1E shows that aging increased the mRNA levels of the major ER chaperone *Grp78* and that of *Pdi*, which play a crucial role in oxidative protein folding and ER homeostasis. Such transcriptional activation of *Grp78* indicates the induction of ER stress in the liver of rats. Because oxidative stress, ER stress, and inflammation are essentially interrelated, we measured the mRNA levels of the pro-inflammatory cytokines *Il-6* and *Tnfα* and the anti-inflammatory cytokine *Il-10* in the liver from both groups of rats. Figure 1F shows that all the cytokines increased their mRNA levels with aging, indicating a state of chronic inflammation and persistent ER and oxidative stress in the liver of aged rats that could be associated with the concentration of circulating CRP shown in Table 1, the accumulation of lipofuscin [15,17], and TBARS (Figure 1A). However, the effects of refeeding, contrary to what was reported [56] but in agreement with our previous observations [15], showed that the mRNA levels of cytokines in the liver were reduced by 30 min of feeding after starvation (Figure 1F). Therefore, the results presented here suggest that the combination of aging and prolonged fasting increases ROS, oxidative stress damage, ER stress, and inflammation in the liver of Wistar rats.

### 3.3. Aging Combined with Prolonged Fasting Perturbed Liver Metabolic Pathways in the Wistar Rat 

**Figure 2:**
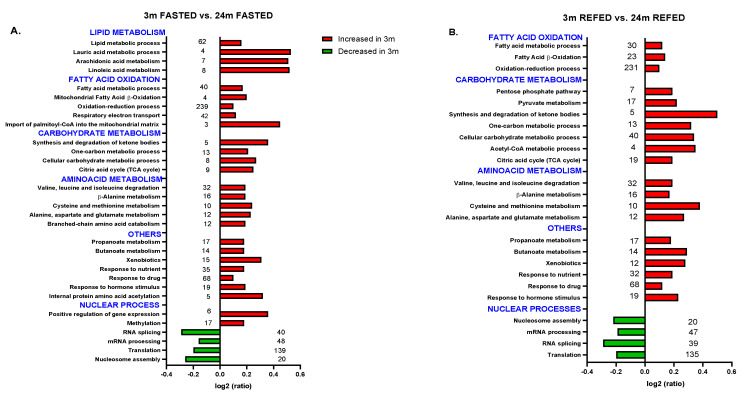
Biological processes and metabolic pathways affected in hepatic NEF of fasted or fasted/refeed rats with aging. Data showed changes in 3- compared with 24-month-old animals. (**A**) Changes after 36 h fasting. (**B**) Changes after 36 h fasting and then refeeding for 30 min. The most representative GOBP and KEGG categories are shown indicating the number of identified proteins per categories. Data are presented as protein log2-ratios between the different experimental groups according to their estimated variances (z_q_ values, see Supplementary Table S4). Red colors represent processes higher in 3- than in 24-month-old rats. Green colors represent processes lower in 3- than in 24-month-old rats.

We further investigated the hepatic NEF proteome to gain insight into the biological processes that take place at the nuclear level related to aging, energy status, and cellular redox balance in Wistar rats. Nuclear enriched proteomes from 3- or 24-month-old rats were analyzed by isobaric labeling followed by LC-MS/MS and compared under a fasting state (Figure 2A) and upon a fasting/refeeding cycle (Figure 2B) to investigate whether nuclear proteomic modulation continued to be observed upon refeeding.

A total of 1686 proteins were quantified in all samples (Supplementary Table S3), and of them 115 proteins were differentially represented after pairwise comparisons between the different groups (FDRq < 0.05) (Supplementary Table S3). Proteins were categorized by biological processes based on their GO BP and KEGG pathway annotations (Supplementary Table S4). Systems biology analysis of the hepatic NEF proteome revealed changes in metabolic and oxidation-reduction processes in old rats (Figure 2A,B). Proteomics data also revealed that in response to the nutritional condition and hormone levels (especially to insulin), several metabolic pathways were decreased in old compared with young rats (Figure 2A,B), particularly the tricarboxylic acid cycle (TCA cycle), fatty acid beta-oxidation, respiratory electron transport, synthesis and degradation of ketone bodies, and drugs and xenobiotics metabolism. Moreover, carbohydrate, fatty acid, amino acid, and butanoate and propanoate metabolic processes were also reduced in old compared with young rats (Figure 2A,B).

Among others, several proteins downregulated by aging are members of the TCA cycle, such as citrate synthase, and members of the OXPHOS complex, such as NADH-ubiquinone oxidoreductase (complex I) and ATP synthase (complex V) (Supplementary Table S4). The Western blot results presented in Figure 1D confirm the above-reported results obtained by proteomics.

### 3.4. Impact of Refeeding after 36 h Fasting in the Nuclear Proteome from Old Wistar Rats

**Figure 3:**
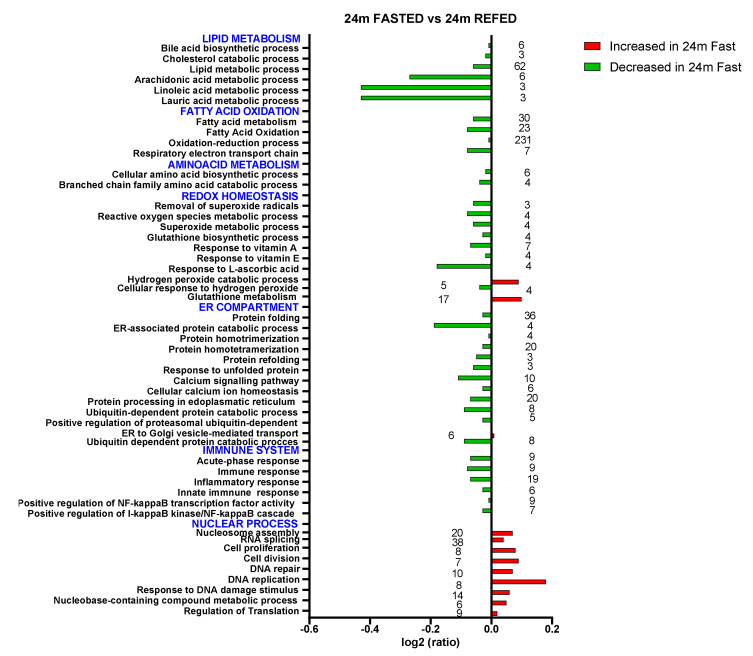
Biological processes and metabolic pathways affected in hepatic NEF of old rats upon refeeding after 36 h of fasting. Changing of biological processes (GOBPs) and metabolic pathways (KEGG and REACTOME) when comparing 24-month fasted vs. 24-month refed rats. The most representative GOBP and KEGG categories are shown indicating the number of identified proteins per categories. Data are presented as protein log2-ratios between the different experimental groups according to their estimated variances (z_q_ values, see Supplementary Table S4). Red colors represent processes higher in 24-month fasted than in 24-month refed rats. Green colors represent processes lower in 24-month fasted than in 24-month refed rats.

Apart from several processes and pathways linked to nutrients and drug metabolism, which were significantly affected in the liver by aging, proteomics results also revealed that refeeding after 36 h of fasting affected biological processes and pathways associated with the cell redox homeostasis and defense against oxidative stress in old rats including removal of superoxide radicals, superoxide metabolic process, cellular response to hydrogen peroxide, glutathione biosynthetic process, and response to L-ascorbic acid, vitamin A and vitamin E, among others, which increased in aged rats upon refeeding (Figure 3). Nevertheless, another set of processes associated with the glutathione metabolic process and response to oxidative stress were reduced in old rats after refeeding (Figure 3). As previously reported [33], most of these changes in old rats were recovered after 30 min of refeeding as compared with age-matched fasted rats (Figure 3).

The main proteins identified related to these processes are shown in the Supplementary Table S4. Among the proteins, we found members of a repertoire of antioxidant systems, including carbonic anhydrase 3, superoxide dismutases, catalase, components of the peroxyredoxin family (1–6), thioredoxin, glutathione transferase, and thiol-containing proteins, confirming the results described in proteomics analysis conducted in the liver of aged Sprague-Dawley rats subjected to prolonged fasting [33]. The reduction of SOD2 confirmed the result presented in Figure 1A related to *Sod2* mRNA levels in the liver.

In addition, most of the upregulated processes and pathways observed in old rats upon refeeding belong to the ER compartment, such as protein folding, ER-associated protein catabolic process, protein homotrimerization, protein homotetramerization, protein refolding, response to unfolded protein, calcium signaling pathway, cellular calcium ion homeostasis, and protein processing in endoplasmic reticulum, as shown in Figure 3.

Moreover, ubiquitin-dependent protein catabolic processes and positive regulation of proteasomal ubiquitin-dependent protein catabolic processes, as part of the unfolded protein response, are upregulated in refed old rats. Nevertheless, protein ubiquitination is downregulated, reinforcing the suggestion that fasting affects protein and lipid homeostasis in the liver of old rats. Additionally, ER to Golgi vesicle-mediated transport is downregulated in refed old rats, suggesting an accumulation of protein in the ER and subsequent ER stress (Figure 3).

These data confirm that the liver of aged rats under prolonged fasting disturbs calcium homeostasis, decreases ER function, and deregulates the ubiquitin-proteasomal system (UPS), which constitutes one of the most important antioxidant systems in cells and which plays a fundamental role in the maintenance of proteostasis, insulin signaling, and hepatic lipid metabolism [57]. In this regard, many age-related pathologies are accompanied by dysregulation of UPS and disturbed proteostasis, such as NAFLD and diabetes [58,59]. Studies investigating the hepatic ER proteome in animal models of type 2 diabetes (*db/db* mice) show severe disruption of the normal functions of the ER in the liver of diabetic mice with great impact on hepatic insulin sensitivity [59].

Supplementary Table S4 shows several proteins involved in the ER process. Among these proteins are GRP78, PDI, Endoplasmin (94 kDa glucose-regulated protein GRP94), NADPH-cytochrome P450 reductase, eukaryotic translation initiation factor 2 subunit 1 (involved in protein kinase RNA-like endoplasmic reticulum kinase (PERK)-mediated regulation of gene expression), the sarcoplasmic/endoplasmic reticulum calcium ATPase 2 (SERCA), as well as several members of the cytochrome P450 family of proteins (Supplementary Table S4).

The results presented in Figure 1A show that TBARS levels were increased in the liver from old rats, which indicates severe oxidative stress damage in aged liver and corroborates the proteomic data. In addition, the upregulation of *Grp78* and *Pdi* observed in the Figure 1E could be understood as an indicator of worsening of ER stress and activation of UPR signaling to restore ER homeostasis, which lead to transcriptional activation of these ER chaperones. In fact, the decrease of the GRP78 protein levels has been considered to cause fat accumulation in livers of mice [60].

Importantly, refeeding after prolonged fasting contributed to an increase in the abundance of proteins known to play a role in the response to oxidative and ER stress in aged Wistar rats. In parallel, we observed that refeeding increases processes associated with the acute phase response, immune response, inflammatory response, innate immune response, positive regulation of NF-kappaB transcription factor activity, and positive regulation of the I-kappaB kinase/NF-kappaB cascade in the liver of old rats (Figure 3).

### 3.5. Aging Combined with Prolonged Fasting Affects Nuclear Processes in Wistar Rats, Particularly RNA Alternative Splicing

We hypothesized that a significant part of aging-mediated liver damage in Wistar rats can be attributed to alterations in gene expression. The NEF proteomic analysis confirmed our hypothesis. In agreement with the role of aging in nuclear genome instability, biological processes and pathways related to regulation of gene expression, such as nucleosome assembly, translation, mRNA processing, and mRNA splicing, were significantly increased in aged rats compared with young rats (Figure 2A). Furthermore, those processes and functions associated with methylation, internal protein amino acid acetylation, and positive regulation of gene expression were reduced in old fasted rats compared with young fasted rats (Figure 2A).

Moreover, in fasted/refed rats, translation, RNA splicing, mRNA processing, and nucleosome assembly were increased in old rats (Figure 2B), suggesting that aging is, in our setting, the main cause that determines the rearrangement of the nuclear proteome, altering the subcellular distribution pattern of several transcription factors previously observed in liver from old Wistar rats [16].

In fact, in the hepatic nuclei of aged rats, regardless of the fasting-refeeding cycle, there was an increase of several histones and nucleosome-interacting proteins, such as nucleophosmin and nucleolin (Supplementary Table S4), indicating age-dependent changes in nucleosome occupancy consistent with previous reports [11]. Furthermore, there was an upregulation of several components of the splicing process, such as the heterogeneous nuclear ribonucleoproteins (hnRNPs) (Supplementary Table S4). Moreover, numerous splicing factors and proteins involved in the control of pre-mRNA splicing, such as the transformer-2 protein homolog beta; several components of the spliceoseome complex, such as the NHP2-like protein 1; the spliceosome RNA helicase Ddx39b; and some serine/arginine-rich splicing factors (SRSFs), were also upregulated (Supplementary Table S4) in the liver of old rats.

Interestingly, many hnRNPs are known to be induced by oxidative stress [61,62]. In addition, various studies in mice have associated the dysregulation of alternative splicing and the altered levels of hnRNPs and SRSFs proteins with the development of cancer [63,64]. Taken together, our findings indicate that aging in Wistar rats could modify the repertoire of the transcriptome and proteome through alternative pre-mRNA splicing, generating different proteins that could modify hepatic cellular function and also contribute to the development of hepatic tumors [24,65]. Moreover, we think that dysregulation of the splicing process that ultimately modulates gene expression could be attributable in part to oxidative stress, as described in [27,28,61,62]. However, the contribution of ER and oxidative stress to changes in the alternative RNA splicing machinery in the liver of old rats need to be further investigated.

Apart from these processes and pathways, DNA duplex unwinding and liver development increased in the liver of old rats after refeeding (Figure 3). Interestingly, in old rats, DNA synthesis and repair, DNA recombination, nucleobase-containing compound metabolic process, cell division, response to DNA damage stimulus, and positive regulation of cell proliferation were reduced compared with young rats under both prolonged fasting and 30 min refeeding as shown in Figure 2A,B and Figure 3, confirming that aging reduces the rates of DNA synthesis and repair, regardless of the fasting-refeeding cycle.

### 3.6. Impact of Refeeding after 36 h Fasting in the Nuclear Proteome from Young Wistar Rats

Interestingly, our proteomic analysis indicates that in 3-month-old rats, 30 min refeeding after 36 h fasting is enough to enhance metabolism and to downregulate some nuclear processes and functions compared to the fasting state, such as those involved in mRNA splicing and spliceosomal complex function. Among the proteins involved are members of the heterogeneous nuclear ribonucleoproteins, the ATP-dependent RNA helicase DDX and the NHP2-like protein 1 (Supplementary Table S4).

In summary, the proteomics data presented here agrees with the data set published previously [62]. More importantly, our data offer an insight into the process of aging in the liver of Wistar rats. Moreover, our results are in agreement with previous data reporting exacerbated oxidative stress in fasting liver [33] and state that aging combined with prolonged fasting weakens the liver´s ability to respond to refeeding. In addition, the recently published temporal nuclear accumulation of proteins and phosphoproteins from mouse liver by SILAC proteomics [66] identified many rhythmic proteins, which were parts of nuclear complexes involved in transcriptional regulation, ribosome biogenesis, DNA repair, and the cell cycle [66].

## 4. Discussion

Many studies have been done to find the link between aging, oxidative stress, and liver damage at the molecular and cellular levels. Although prolonged fasting and periodic fasting cycles have shown efficacy for weight loss and have profound beneficial effects on many different indexes of health in rodents and humans [31,67], prolonged fasting could exert adverse effects in aged organisms with multiple age-related diseases. Here, we analyzed the possible pathways accounting for liver steatosis in Wistar rats and the role of aging combined with prolonged fasting and oxidative stress in these mechanisms. Hepatic lipid accumulation, originating from deregulated de novo lipogenesis and fatty acid oxidation, is the principal factor that enhances the transition from normal liver to steatosis, steatohepatitis, fibrosis, cirrhosis, and eventually hepatic carcinoma [68]. Several factors like insulin resistance, obesity, and age contribute to hepatic lipid accumulation [6,7]. The exact role of aging in hepatic steatosis is not entirely clear.

Research has suggested that aging increases oxidative stress damage of cellular components, reduces the ability of the liver to inactivate toxins, induces ER stress and inflammation, impairs proteostasis, and alters cellular structure of hepatocytes and their metabolism [65]. Moreover, the aged liver also manifests alterations of the genome and epigenome, as part of all cellular hallmarks of aging [69].

Currently, most of our knowledge about the molecular changes that occur in the liver of Wistar rats with aging comes from studies of gene expression patterns. The nucleus is the site of control of gene expression and accumulated evidence indicates that aging induces structural and functional changes in the nucleus that affect the aging process [70]. In this regard, histone modifications, changes in DNA methylation profiles, and nuclear accumulation of factors, such as cyclin-dependent kinase 4 (cdk4), with ageing promotes NAFLD and increases the severity of the disease [71,72]. Although some recent studies suggest that splicing variants may play a role in NAFLD development [22,24,25,73], liver-specific alternative splicing and their significance in the process of fatty liver disease in aged rats is less well understood. Related to this, there is a report associating a change in gene organization within the chromatin and a decrease in hepatocytes proliferation with a less dynamic association of the chromatin with the nuclear matrix in Wistar rat livers upon aging [74]. Thus, these findings could potentially explain the changes observed in our work related to alternative splicing, mRNA processing, and nucleosome assembly.

In this work, we focused on the hepatic NEF proteome with the aim of exploring the organelle processes that explain the age-related changes that affect the development of NAFLD in Wistar rats. Second, and to emphasize the implication of oxidative stress and inflammation, we analyzed the adaptation of the liver to prolonged fasting, an intervention that increases oxidative stress in this organ [29,30,33].

Our findings are consistent with previous knowledge in rodents [30,60,63,65]. The proteomics analyses highlighted those proteins that are involved in macronutrients metabolism, drug metabolism (a function of smooth ER), and oxidative phosphorylation OXPHOS (a mitochondrial process) were globally downregulated in older rats under prolonged fasting compared with young rats in the same condition. Concerning the metabolic process, oxidation-reduction process, response to drugs, fatty acid beta oxidation, ketone bodies synthesis, and degradation were significantly altered in aged liver, increasing our understanding of impaired hepatic metabolism of nutrients and drugs in older organisms under prolonged fasting.

Regarding the response to oxidative stress, several proteins were downregulated in livers from old rats due to the combination of aging and prolonged fasting, especially those associated with antioxidant functions, confirming that nutrient deprivation impairs the antioxidant capacity of the liver in old animals, enhancing oxidative damage [29,30,33,75]. In fact, our results also indicated that prolonged fasting induces a significant increase in fat storage and fat peroxidation in the liver of old rats compared with their younger counterpart.

Interestingly, the proteomic analysis did not reveal any change in processes associated with the oxidative stress response in young rats under prolonged fasting or upon the fasting/refeeding cycle. Many studies have reported that periodic fasting, intermittent fasting, and caloric restriction reduces the metabolic alterations accumulated over time, protecting against aging and disease and increasing the lifespan or health span in mammals [67,76,77]. In this regard, published reports suggest that fasting regimes as food restriction improve MS parameters and reverse the hepatic features of NAFLD [76,77]. Nevertheless, aging combined with prolonged fasting exacerbated steatosis in rats. Thus, we suggest that prolonged fasting is detrimental for older animals with MS and NAFLD.

Importantly, our findings indicate that proteins that are involved in alternative splicing, spliceosome components, and nucleosome assembly were upregulated in nuclear liver from old compared with young rats, regardless of the prolonged fasting-refeeding cycle, indicating that dysregulation of the splicing process is present in the liver of old rats with NAFLD as in humans [22].

Alternative splicing of pre-mRNAs is a major process contributing to transcriptome diversity in higher eukaryotes. Because most of the altered mRNAs or splicing factors described until now in steatotic livers are associated with lipid metabolism [78], we suggest that changes in RNA splicing are among the alterations that disturb lipid metabolism in liver from old Wistar rats under prolonged fasting and may worsen NAFLD and other more serious liver diseases.

To the best of our knowledge, this is the first work that has compared the hepatic nuclear proteome profile of young and old Wistar rats. The results presented here provide a comprehensive molecular basis of aged liver responses when facing a major energetic challenge. Moreover, in the absence of the best-suited animal models of NAFLD that develop in their entirety the human disease, the aged Wistar rat appears to mimic the progression of NAFLD with aging. In this regard, old Wistar rats manifest mild obesity with increased visceral adiposity, dyslipidemia, insulin resistance, systemic inflammation, and liver steatosis with mild perisinusoidal fibrosis, in which the nucleo-cytoplasmic transport of several transcription factors is impaired and the lipogenic capacity is increased [15,16,17,18]. As it has been previously described, all these conditions are associated with increased oxidative stress and ER stress [6,58]. Finally, the proteomics results highlight reduced ER function and oxidative stress response in the liver of old Wistar rats and point to alternative splicing as an important mechanism of change of liver functions. Therefore, the aging Wistar rat could be an attractive model to study the molecular basis of the progression of NAFLD during physiological aging.

## 5. Conclusions

In summary, quantitative comparative analysis of the hepatic nuclear proteome revealed that several biological processes of the nucleus are disrupted in the liver of old Wistar rats, regardless of nutritional status, leading to enhanced RNA processing and alternative splicing and reduced capacity for DNA repair and nucleocytoplasmic transport. Further research is needed to understand the interdependent relation between aging, oxidative stress, and dysregulation of the splicing process in the decline of liver function during aging combined with prolonged fasting.

## Figures and Tables

**Table 1:** Serum and liver metabolic parameters in 3- and 24-month-old rats in response to fasting or fasting/refeeding.

	3m		24m		2-way-ANOVA		
	36 h Fasting	36 h Fast + 30 min Refeed	36 h Fasting	36 h Fast + 30 min Refeed	Young vs. Old	Fast vs. Refeed	Interaction
Liver TAG (mg/g)	4.7 ± 0.8	3.4 ± 0.4	12.7 ± 2 *	12.4 ± 1	*p* < 0.0001	*p* = 0.5361	*p* = 0.6998
Liver Glycogen (mg/g)	2.0 ± 0.008	4.0 ± 0.3 ^++++^	4.9 ± 0.1 ****	5.7 ± 0.2	*p* < 0.0001	*p* < 0.0001	*p* = 0.0376
Serum glucose (mM)	4.9 ± 0.8	6.1 ± 0.5 ^++^	5.12 ± 0.4	5.6 ± 0.4	*p* = 0.6141	*p* = 0.0043	*p* = 0.0762
Serum TAG (mg/dL)	29 ± 2	33 ± 4	52 ± 5 *	57 ± 4	*p* = 0.0003	*p* = 0.3750	*p* = 0.9387
Serum NEFA (mm/L)	0.58 ± 0.04	0.52 ± 0.06	0.55 ± 0.03	0.97 ± 0.1 ^++^	*p* = 0.0215	*p* = 0.0465	*p* = 0.0106
Serum TKB (mm/L)	2.3 ± 0.1	0.18 ± 0.06 ^++++^	1.48 ± 0.1 *	0.34 ± 0.06 ^++^	*p* = 0.0174	*p* < 0.0001	*p* = 0.0016
Serum insulin (ng/mL)	0.71 ± 0.2	2.73 ± 0.1 ^++^	2.5 ± 0.1 **	2.39 ± 0.2	*p* = 0.0069	*p* = 0.0021	*p* = 0.0008
Serum glucagon (pg/mL)	318 ± 9	355 ± 6	538 ± 14 **	251 ± 19 ^++++^	*p* = 0.0039	*p* < 0.0001	*p* < 0.0001
Serum leptin (ng/mL)	1.5 ± 0.06	1.4 ± 0.2	4.9 ± 0.5 **	4.6 ± 0.84	*p* < 0.0001	*p* = 0.5402	*p* = 0.9577
Acetylated ghrelin (ng/mL)	0.13 ± 1.9	0.13 ± 1.3	0.23 ± 1.8 **	0.18 ± 2.4	*p* = 0.0005	*p* = 0.1968	*p* = 0.1337
Nonacetylated ghrelin (ng/mL)	1.26 ± 0.2	1.24 ± 0.1	0.8 ± 0.03	0.7 ± 0.03	*p* = 0.0045	*p* = 0.6772	*p* = 0.7902
Acetylated/nonacetylated ghrelin ratio	0.12 ± 0.06	0.11 ± 0.01	0.29 ± 0.02 **	0.26 ± 0.04	------	------	------
Serum ALT (IU/L)	5.01 ± 0.8	6.6 ± 0.4	12.0 ± 1 *	15.1 ± 1	*p* < 0.0001	*p* = 0.1240	*p* = 0.6111
Serum CRP (µg/mL)	209 ± 1	212 ± 35	463 ± 12 ****	382 ± 9	*p* < 0.0001	*p* = 0.0412	*p* = 0.2369

Effects of fasting (36 h) or fasting (36 h) and then refeeding (30 min) on liver TAG, serum glucose, triacylglycerides (TAG), non-sterified fatty acids (NEFA), total ketone bodies (TKB), insulin, glucagon and leptin, plasma acetylated and nonacetylated ghrelin and the acetylated/nonacetylated ghrelin ratio in plasma, serum alanine aminotransferase (ALT) and C-reactive protein (CRP) in young (3 m) and old (24 m) Wistar rats. Results are the mean ± SEM of 4 rats per group. Data were analyzed by Two-way ANOVA followed by Tukey’s correction. Two-way ANOVA was performed to detect main effects of age, fasting-refeeding, and the interaction. * *p* < 0.05, ** *p* < 0.01, **** *p* < 0.0001 vs. the young rats. ^++^ *p* < 0.01, ^++++^ *p* < 0.0001 vs. the age-matched fasted rats.

## Data Availability

All relevant data are included within the manuscript and in the Supplementary Materials.

## Supplementary Materials

The following are available online at https://www.mdpi.com/article/10.3390/antiox10101535/s1, Table S1: Probes used for real time PCR. Table S2: Serum and liver metabolic parameters in 3- and 24-month-old Wistar rats killed after a 16 h and 36 h fast. Table S3: Proteins quantified in nuclear enriched fraction (NEF) from 3-month-old and 24-month-old Wistar rat. Table S4: Biological processes and metabolic pathways altered in rat liver nuclear enriched fractions (NEF) upon aging or fasting-refeeding cycle. Representative categories affected (FDRc ≤ 0.05%) are shown, indicating their corresponding identified proteins, their standardized quantitation (zq) shaded according to a color scale shown at the top and the number of peptides per protein detected. The GO terms and KEGG pathways were distributed into several pathways and functions, including tricarboxylic acid cycle (TCA), electron transport chain and ATP synthesis, ER overload response, response to oxidative stress and acute phase response, as well as nuclear-specific pathways and functions such as DNA synthesis, DNA damage and repair, RNA processing and splicing, nucleosome assembly and chromatin remodeling, among others. Figure S1: An overview of the methods and procedures employed in this work.

## Abbreviations

ALT: alanine aminotransferase; CPT-1: carnitine-palmitoyl transferase-1; CRP: C-reactive protein; Cyp2c11: Cytochrome P450 monooxygenase isoforms 2c11; ChREBP: carbohydrate-responsive element-binding protein; DGAT: diacylglycerol acyltransferase; DNA: deoxyribonucleic acid; ELISA: enzyme-linked immunosorbent assay; ER: endoplasmic reticulum; Fmo3: Flavin Containing Dimethylaniline Monoxygenase 3; Foxo1: Forkhead Box O1; Foxa2: Forkhead box A2; GOBP: Gene ontology biological processes; Grp78: 78-kDa glucose-regulated protein; HDL: high-density lipoprotein; HFD: high fat diet; HNF4α: Hepatocyte nuclear factor 4 alpha; hnRNP: Heterogeneous nuclear ribonucleoproteins; Il-6: Interleukin 6; Il-10: Interleukin 10; iTRAQ: isobaric tag quantitation; KEGG: Kyoto Encyclopedia of Genes and Genomes; mRNA: messenger ribonucleic acid; MTTP: microsomal triglyceride transfer protein; MS: metabolic syndrome; MUFAs: monounsaturated fatty acids; NAFLD: non-alcoholic fatty liver disease; NASH: non-alcoholic steatohepatitis; NEF: nuclear enriched fraction; NEFA: nonesterified fatty acid; OXPHOS: mitochondrial oxidative phosphorylation system; Pdi: Protein disulfide isomerase; ROS: Reactive oxygen species; Scd-1: Stearoyl-CoA desaturase 1; Sod2: Superoxide dismutase 2; SRSFs: Serine/arginine-rich splicing factors; TAG: triacylglycerides; TBARS: Thiobarbituric acid reactive substances; TCA: tricarboxilic acid cycle; T2D: type 2 diabetes mellitus; TKB: total ketone bodies; Tnfα: Tumor necrosis factor alpha; UPR: unfolded protein response.
