# Supplementary material for: Aging Induces Hepatic Oxidative Stress and Nuclear Proteomic Remodeling in Liver from Wistar Rats
Source: Antioxidants (Basel). 2021 Sep 27;10(10):1535. doi: 10.3390/antiox10101535 (PMC8533122; doi:10.3390/antiox10101535)
Supplement: Supplementary file 1 [file antioxidants-10-01535-s001.zip › 21.9.21Frvs Supplementary Material.pdf]

Supplemental Material

Details about the genes used in this study are provided in Supplemental Table S1.

Supplemental Table S1. Probes used for real time PCR.

Gene	ABI Assay ID
Sod2	Rn99999088_g1
Scd-1	Rn00594894-g1
Fmo3	Rn00584825_m1
Cyp2c11	Rn01502203_m1
Il-6	Rn00561420_m1
Il-10	Rn00563409_m1
Tnf-α	Rn99999017_m1
Grp78	Rn01435769_g1
Pdi	Rn00564459_m1
18S rRNA	4319413E

Supplemental Table S2. Serum and liver metabolic parameters in 3- and 24-month-old Wistar rats killed after a 16h and/or 36h fast

	3m		24m		2-Way-ANOVA		
Fasting	16 hours	36 hours	16 hours	36hours	Young vs. old	16h vs. 36h	Interaction
Body Weight (g)	325±7	329±7	594±19*	572±22	p<0.0001	p=0.5271	p=0.4496
Insulin (ng/mL)	1.3±0.2	0.71±0.2$^+$	1.4±0.2	2.5±0.1$^+$	p=0.0002	p=0.1826	p=0.0005
Glucose (mM)	4.5±0.2	4.9±0.8	5.0±0.1	5.12±0.4	p=0.4624	p=0.5975	p=0.7505
NEFA (mm/L)	1.03±0.02	0.58±0.04$^+$	1.08±0.03	0.55±0.03$^+$	p=0.7512	p<0.0001	p=0.2188
TKB (mM)	0.72±0.09	2.3±0.1$^+$	0.31±0.01*	1.48±0.1$^+$	p<0.0001	p<0.0001	p=0.0311
Liver weight (g)	10.5±0.6	8.7±0.2$^+$	18.9±5*	12.1±0.5$^+$	p=0.0381	p=0.1153	p=0.3430
Liver TAG (mg/g)	6.1±0.3	4.7 ± 0.8	9.5±0.7*	12.7±2$^+$	p=0.0003	p=0.4461	p=0.0671
Liver TBARS (nmoles/mg protein)	0.13±0.02	0.45 ± 0.05^{++}	0.34±0.07**	1.52±0.04^{+++}	p<0.0001	p<0.0001	p<0.0001
Visceral fat (g)	9.8±2	10.1 ± 0.3	35.7±3*	36 ± 1	p<0.0001	p=0.8757	p=0.9891

Results are the mean ± SEM of 4 rats per group. Data were analyzed by Two-way ANOVA followed by Tukey's correction. Two-way ANOVA was performed to detect

main effects of age, time of fasting (16h vs. 36h), and their interaction. Results with values of $p < 0.05$ were considered statistically significant. *p<0.05, **p<0.01, *vs* the young rats. $^{+}$p<0.05, $^{++}$p<0.01, $^{+++}$p<0.001 *vs* the age-matched rats

Supplemental Table S3. Proteins quantified in nuclear enriched fraction from 3-month- and 24-month-old Wistar rats. F: 36h fast; F+R: 36h fast + 30 min refed. The Excel tables are available in the compressed zip file named Supplemental Material

Supplemental Table S4. Biological processes and metabolic pathways altered in rat liver nuclear enriched fractions upon aging or fasting-refeeding cycle. Representative categories affected (FDRc≤0.05%) are shown, indicating their corresponding identified proteins, their standardized quantitations (z_q) shaded according to a color scale shown at the top and the number of peptides per protein detected. F: 36h fast; F+R: 36h fast + 30 min refed. The Excel tables are available in the compressed zip file named Supplemental Material

Supplemental Figure S1. An overview of the methods and procedures employed in this work. The figure is available in the compressed zip file named Supplemental Material
