# Supplementary figures and images for: Aging Induces Hepatic Oxidative Stress and Nuclear Proteomic Remodeling in Liver from Wistar Rats
Source: Antioxidants (Basel). 2021 Sep 27;10(10):1535. doi: 10.3390/antiox10101535 (PMC8533122; doi:10.3390/antiox10101535)

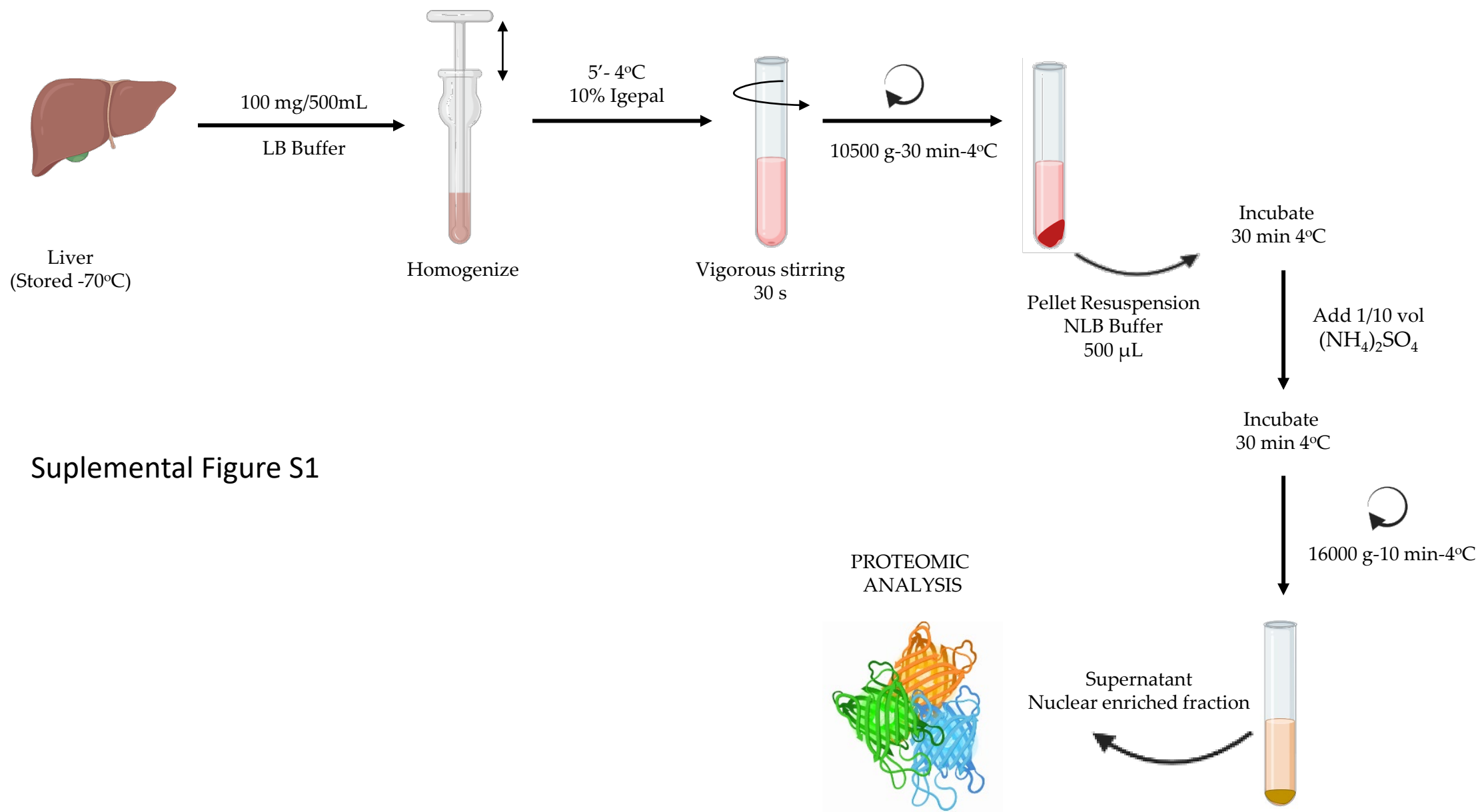

Supplemental Figure S1

Supplement: Supplementary file 1 [file antioxidants-10-01535-s001.zip › Figure S1.pdf]
